# WildDrone: autonomous drone technology for monitoring wildlife populations

**DOI:** 10.3389/frobt.2025.1695319

**Published:** 2026-01-12

**Authors:** Ulrik Pagh Schultz Lundquist, Saadia Afridi, Clément Berthelot, Nguyen Ngoc Dat, Kasper Hlebowicz, Elena Iannino, Lucie Laporte-Devylder, Guy Maalouf, Giacomo May, Kilian Meier, Constanza A. Molina Catricheo, Edouard G. A. Rolland, Camille Rondeau Saint-Jean, Vandita Shukla, Tilo Burghardt, Anders Lyhne Christensen, Blair R. Costelloe, Matthijs Damen, Andrea Flack, Kjeld Jensen, Henrik Skov Midtiby, Majid Mirmehdi, Fabio Remondino, Tom Richardson, Benjamin Risse, Devis Tuia, Magnus Wahlberg, Dylan Cawthorne, Steve Bullock, William Njoroge, Samuel Mutisya, Matt Watson, Elzbieta Pastucha

**Affiliations:** 1 SDU UAS, Mærsk Mc-Kinney Møller Institute, University of Southern Denmark, Odense, Denmark; 2 Avy, Amsterdam, Netherlands; 3 Department of Migration, Max Planck Institute of Animal Behavior, Konstanz, Germany; 4 Department of Biology, University of Konstanz, Konstanz, Germany; 5 School of Computer Science, University of Bristol, Bristol, United Kingdom; 6 Department of Environmental Sciences, Wageningen University and Research, Wageningen, Netherlands; 7 Department of Collective Behavior, Max Planck Institute of Animal Behavior, Konstanz, Germany; 8 Department of Biology, University of Southern Denmark, Odense, Denmark; 9 Environmental Computational Science and Earth Observation Laboratory, Environmental Engineering Institute, École Polytechnique Fédérale de Lausanne (EPFL), Sion, Switzerland; 10 School of Civil, Aerospace and Design Engineering, University of Bristol, Bristol, United Kingdom; 11 Institute for Geoinformatics and Faculty of Mathematics and Computer Science, University of Münster, Münster, Germany; 12 3D Optical Metrology (3DOM), Bruno Kessler Foundation (FBK), Trento, Italy; 13 Centre for the Advanced Study of Collective Behaviour, University of Konstanz, Konstanz, Germany; 14 Ol Pejeta Conservancy, Nanyuki, Kenya; 15 School of Earth Sciences, University of Bristol, Bristol, United Kingdom

**Keywords:** biodiversity conservation, conservation ecology, autonomous drones, computer vision, wildlife monitoring

## Abstract

The rapid loss of biodiversity worldwide is unprecedented, with more species facing extinction now than at any other time in human history. Key factors contributing to this decline include habitat destruction, overexploitation, and climate change. There is an urgent need for innovative and effective conservation practices that leverage advanced technologies, such as autonomous drones, to monitor wildlife, manage human-wildlife conflicts, and protect endangered species. While drones have shown promise in conservation efforts, significant technological challenges remain, particularly in developing reliable, cost-effective solutions capable of operating in remote, unstructured, and open-ended environments. This paper explores the technological advancements necessary for deploying autonomous drones in nature conservation and presents the interdisciplinary scientific methodology of the WildDrone doctoral network as a basis for integrating research in drones, computer vision, and machine learning for ecological monitoring. We report preliminary results demonstrating the potential of these technologies to enhance biodiversity conservation efforts. Based on our preliminary findings, we expect that drones and computer vision will develop to further automate time consuming observational tasks in nature conservation, thus allowing human workers to ground conservation actions on evidence based on large and frequent data.

## 1 Introduction

Our planet is currently undergoing an exceptionally rapid loss of biodiversity, with more species threatened with extinction now than at any other point in human history (Ceballos et al., 2017). The key drivers of this loss of biodiversity include climate change, over-exploitation, and habitat destruction due to agricultural expansion, urbanization, and land degradation (Veach et al., 2017; Dinerstein et al., 2019). Given the urgency of the situation, there is a critical need for effective and practical nature conservation practices for monitoring wildlife populations, tracking animal movement, and managing human-wildlife conflicts.

Recent technological advances have opened up new possibilities for more precise and large-scale interventions to prevent declines in wildlife populations. Among these advances, robotic solutions such as drones (unmanned aerial vehicles, UAVs, part of an unmanned aircraft system, UAS) have proven to be effective tools for observation tasks (Hodgson et al., 2016): drones can capture data over larger areas, at higher frequency and at a significantly reduced cost compared to more conventional methods. Drone-based imaging enables mesoscale data acquisition and therefore bridges the gap between large-scale (e.g., satellite imagery) and ground-level data collection (e.g., camera traps). In fact, mesoscale recordings offer an optimal trade-off for monitoring macrofauna and are therefore of great importance for wildlife monitoring (Linchant et al., 2015). Moreover, drones enable monitoring of species in complex environments, including areas that are difficult for humans to access (Kellenberger et al., 2021) and extreme environments (Pina and Vieira, 2022; Pfeifer et al., 2019), allowing researchers to track populations and individuals at unprecedented resolutions (Xue et al., 2021; Wu et al., 2023).

For drone technology to be used effectively in nature conservation, it must be reliable, cost-effective, user-friendly, and capable of operating in remote, unstructured, open-ended environments with minimal infrastructure. Currently, drones lack the technological maturity for widespread application in this field; they are often viewed as unreliable, time-consuming, and expensive for use in ecological experiments and conservation efforts. There is thus a pressing need for the development of robust, predictable, and affordable drone technologies. Additionally, there is a mismatch between the data that can be acquired with drones (images or videos) and our ability to process it and convert it into ecological insights. Data collection volume is being drastically increased by drones and other advanced sensor technologies, which have propelled ecology into the era of big data (Linchant et al., 2015; Farley et al., 2018). Approaches to efficiently convert the data acquired by drones into useful information to address ecological challenges are more necessary than ever.

The combination of drones and computer vision offers significant opportunity for impact. New computer vision techniques applied to drone-based wildlife imagery can significantly contribute to achieving the United Nations Sustainable Development Goals (SDGs) by enhancing our ability to monitor and protect biodiversity. By automating the detection and classification of species, these techniques improve the efficiency and accuracy of wildlife population assessments, aligning with SDG 15 (Life on Land) to halt biodiversity loss. Furthermore, such advancements facilitate the early detection of threats like habitat destruction or poaching, aiding in conservation strategies and promoting SDG 13 (Climate Action) by ensuring ecosystems remain resilient. Additionally, improved wildlife monitoring supports local communities dependent on natural resources, contributing to SDG 1 (No Poverty) and SDG 8 (Decent Work and Economic Growth) by fostering sustainable ecotourism and resource management.

The development of technology suitable for autonomous conservation missions presents a significant technological challenge. Such systems must meet the needs of wildlife researchers and conservation practitioners and must incorporate automated data interpretation through computer vision and machine learning to generate actionable biological and ecological insights. Developing these automated systems necessitates a continuous development cycle that involves domain experts from various fields (drones, computer vision, and conservation ecology) working collaboratively to define requirements and devise solutions. Interdisciplinary collaboration between academia and industry is essential to address the challenge of developing robust and applicable technologies for nature conservation.

The WildDrone project is an EU-funded MSCA Doctoral Network that developes autonomous drone technologies for nature conservation (Lundquist, 2024). The goal of the network is to develop effective and reusable technologies and methodologies that support ecological research and conservation practices. Central to WildDrone is the integration of ecological science with technological development, ensuring that new technologies are continuously shaped by and evaluated against real-world conservation needs. Two primary challenges are targeted: (i) achieving safe and adaptable autonomous drone operations, and (ii) automating data processing through advanced computer vision techniques. By addressing these challenges, WildDrone aims to significantly enhance the utility of drones in conservation by automating time-consuming tasks. The project emphasizes interdisciplinary training for researchers across Europe and Africa, leveraging the diverse expertise of its partners to foster innovation in aerial robotics, computer vision, and wildlife ecology. Central to this effort is a network of thirteen doctoral candidates (DCs) working across three interconnected research themes: Theme 1 - Automated Wildlife Tracking for Conservation (5 DCs), Theme 2 - Safe and Flexible Drone Operations (4 DCs), and Theme 3 - Effective Computer Vision for Conservation (4 DCs). Ultimately, WildDrone seeks to revolutionize wildlife conservation by developing practical tools for monitoring wildlife populations, behaviours, and habitats, and by exploring the trade-offs between using low-cost drones for community science versus more advanced systems for specialized applications. This approach will provide a range of solutions tailored to different conservation needs, promoting broader adoption of these innovative technologies.

The paper is structured as follows. In Section 2, we derive the requirements for a drone-based monitoring system for the conservation of wildlife. In Section 3, we present the WildDrone methodology to develop systems that meet these requirements. In Sections 4, 5, 6, we respectively present specific challenges and results related to automated wildlife tracking, safe drone operation, and computer vision for conservation practice. Finally, in Section 7, we provide concluding remarks.

## 2 Drone-based monitoring of wildlife populations

Research in WildDrone is structured around cross-theme use cases, which form *collaboration clusters* within the network. Each cluster begins with a problem or research question rooted in conservation ecology, where ecological needs and challenges define the data and methodological requirements. These requirements then guide the development of drone operations and computer vision technologies, ensuring that technical innovations are directly aligned with ecological goals. Conservation ecology not only motivates these developments but also contributes critical datasets—such as imagery and tracking data—and domain expertise that inform hardware and software design. In turn, advancements in aerial robotics and computer vision provide novel tools and methods that expand the possibilities for ecological research and conservation practice. This iterative and collaborative process ensures that technological innovation is both ecologically relevant and scientifically impactful.

### 2.1 Motivation: conservation ecology

As human populations expand into natural areas, conflicts between people and animals increase. Human-initiated conflicts, such as poaching and noise pollution, are major threats to species around the world (Linkie et al., 2003; Ferreira et al., 2015; Weilgart, 2007). Anti-poaching efforts are limited by the ability of security teams to efficiently patrol vast protected areas to detect and apprehend poachers (Mulero-Pázmány et al., 2014; de Knegt et al., 2021). Anthropogenic marine activities, such as drilling and construction, can negatively impact marine fauna over vast areas (Weilgart, 2007). Other conflicts, such as livestock predation, negatively impact human livelihoods, engender negative attitudes toward wildlife, and prompt retaliatory killings (Woodroffe et al., 2005; Treves et al., 2007).

To address these challenges, there is a critical need for automated systems capable of monitoring the movement and behaviour of wildlife, which would enable conservation practitioners to mitigate conflicts more effectively. In concrete WildDrone use cases, drones will be deployed and assessed as tools for indirectly detecting poachers by monitoring wildlife behavioural responses to human presence; determining when sensitive marine wildlife is present in areas with construction activities; predict predator movement and hunting behaviour to reduce conflict with livestock; and aid conservation practitioners in locating and identifying rhinoceros in wildlife reserves. These applications highlight the potential of drone technology to serve as a versatile tool for predicting and preventing conflicts between wildlife and humans.

Recent advances in animal-mounted tracking devices have revolutionized ecologists’ ability to collect movement data on a wide range of animal species as they traverse landscapes, continents, and hemispheres (Kays et al., 2015). To interpret this data, researchers need to understand the environmental and social context of animal movement behaviour. Secondary sensors, such as cameras, altimeters, and thermometers can provide contextual data but are often heavy and resource-intensive, and therefore unsuitable for mounting on many wildlife species (Williams H. et al., 2020). Drones can be flexibly deployed to gather data on terrain, land use, and environmental factors such as wind and thermal uplifts, as well as on animals themselves and their interactions (Anderson and Gaston, 2013; Graving et al., 2019). Drones have previously been combined with animal tracking to document environmental conditions along avian foraging paths (Rodríguez et al., 2012; Wilmers et al., 2015), compare the quality of ungulate foraging habitats (Scheiger et al., 2015), and monitor deforestation in primate home ranges (Rodríguez et al., 2012). So far, applications have relied heavily on visual sensors, and researchers have manually converted animal movement paths into flight missions.

Incorporating a wider range of sensors into drone-based missions and developing automated means of integrating bio-logging tags and drone operating systems will allow for more efficient collection of a wider range of relevant contextual data. In concrete WildDrone use cases, drones will be used to monitor the behaviour of prey in response to tagged predators, to determine the air conditions and landscape cues that affect the flight paths of migratory birds, and to perform automated real-time wildlife observations with minimal risk of behavioural interference.

### 2.2 Technology: autonomous drones

Autonomous drones are a promising technology with great potential for adaptive and flexible monitoring of animals in their natural environment. However, current systems do not offer the combination of long endurance Beyond Visual Line Of Sight (BVLOS) operations; Vertical Take-Off and Landing (VTOL); and the ability to capture close-up images of individual animals from multiple, multi-angle view-points. VTOL could for example be combined with onboard navigation and re-routing to enable operations in remote locations with complex terrains.

Operations with multiple drones are seen as key to widening the geographical and temporal coverage of drone missions. However, the complexity and variability of missions required for nature conservation implies a need for dedicated planning and control algorithms to be developed for each specific scenario, which is tedious and time consuming (Campusano et al., 2020). Moreover, the ability to precisely geolocate animals observed by drones is crucial for subsequent analysis of the data collected (Koger et al., 2023).

To fully make use of the benefits that BVLOS operations offer, local re-routing for terrain avoidance needs to be combined with mission-level planning and control algorithms which are scenario-specific. These are also the key to widening the geographical and temporal coverage of drone missions. EU expects that widespread use of BVLOS operations in non-controlled airspace, such as required for the kind of nature conservation effort we envision, will be based on the use of UTM (UAS traffic management) to safely guide drones (SESAR-JU, 2017). To operate within a UTM framework, on-board guidance systems would need to be integrated with UTM systems suitable for the adaptive and flexible BVLOS nature of conservation missions, both in Europe and Africa. Moreover, one of the key requirements for the mission system will be the need for low-impact operations, with the physical aircraft design enabling the creation of drones that are unobtrusive towards the animals being observed when deployed for the purposes of conservation.

In low-income and resource-limited contexts, affordability must be paired with environmental robustness and local maintainability. Field deployments in hot, strong sunlight, dusty, and windy settings often expose lightweight UAS to pre-flight thermal loading, particulate ingress, and handling stresses, which argues for simple thermal management (e.g., ventilated fuselages or directed airflow during cruise), sealed or baffled electronics bays, and conservative airframe margins (Duffy et al., 2018). To enable on-site repair by non-specialists, designs that prioritize modular airframes, avionics using standard connectors, and predominantly off-the-shelf components reduce downtime and reliance on specialized tooling are preferred (Paneque-Gálvez et al., 2014; Mesquita et al., 2021). More broadly, scientific reviews of conservation UAS emphasize that addressing such operational and maintenance considerations is important for sustained uptake in protected areas (Jiménez López and Mulero-Pázmány, 2019).

### 2.3 Technology: computer vision

Machine learning and computer vision deal with learning patterns from data (Hastie et al., 2001) and are becoming more and more prevalent in ecology (Tuia et al., 2022). Supervised approaches, where a learning algorithm is trained on input (the drone images) and output (the quantity to be predicted) pairs, are increasingly used in ecology: approaches based on deep learning—a family of machine learning methods based on artificial neural networks—are promising for connecting the dots between the data acquired and ecological insights (Christin et al., 2019; Kwok, 2019a; b).

Drones can be used to understand how groups of animals are structured, how they move and interact in complex ecosystems. Conservation efforts require knowledge of the numbers and location of animals and of the interaction of individual animals with their habitat. Thus, effective conservation drone systems require automation for large-scale assessment of animal locations and numbers (censuses). For a long time, drone-based animal censuses were predominantly carried out through manual photo interpretation, which is costly, time-consuming, and challenging, in part due to the heterogeneous distribution of animals on the landscape and high terrain variability in aerial images (Kellenberger et al., 2018). Computer vision approaches aiming at automating censuses across geographical areas are urgently needed, and first approaches are appearing: initially, the methods proposed produced many false positives which required further human review (Nicolas et al., 2017), but recent methods have been shown to maintain high detection rates with significantly fewer false positives (Kellenberger et al., 2018; Delplanque et al., 2022; Hoekendijk et al., 2021) and to match, if not surpass, *in-situ* photo-based surveys, while reducing massively survey time and annotation costs (Delplanque et al., 2023). In parallel, community engagement and software tools are appearing (Kellenberger et al., 2020) for providing label information to train such models, information that can be obtained by citizen science and crowdsourcing. Being able to detect, count and characterize animals (e.g., by their species) is a significant step towards population modelling that can then be used to study the interactions of animals.

Assessment of animal behaviour (as outlined in Section 2.1) requires the precise delineation of animal movement trajectories of herds, i.e., multiple animal tracking (Koger et al., 2023), but also the obtention of precise biometric characteristic of animals (posture, size, etc.) (Andrew et al., 2017). To obtain this information, being able to control the drone flight plan in almost real time is essential. But to be able to change the flight plan according to specific animals and remarkable features of interest being observed, an onboard vision system is required, so that the drone can fly closer to an animal to take the necessary images for identification and animal biometrics (Andrew et al., 2020). For autonomous systems, such manoeuvres require real-time on-board navigation based on dynamic tracking data from animals. This way, the end user would receive the images that are needed rather than those that are given by a pre-defined flight plan. Once these images (close ups, tracking of specific individuals showing characteristics of interest) are obtained, one can work on detecting identity (Crall et al., 2013; Brust et al., 2017; Stennett et al., 2022) and other non-behavioural biometrics such as animal posture and size (Nath et al., 2019), the level of alert, or sex and age of the animal under monitoring (Freytag et al., 2016).

Beyond tracking and identifying individual animals, autonomous monitoring of wildlife also requires quantifying their behaviour within the spatial and environmental context of their natural habitat, which is fundamental to interpreting these behaviours in an ecological framework (Haalck et al., 2020). Moreover, ecological factors, conservation management and animal welfare considerations also benefit from quantitative descriptions of the surrounding environment (Greggor et al., 2019; Bracke and Hopster, 2006). Therefore, habitat reconstructions are pivotal and have to be integrated into the former mentioned detection and tracking strategies (Haalck et al., 2023). Image-based 3D reconstructions of natural habitats provide a powerful tool, enabling detailed and accurate models of environments essential for studying species and ecosystems (Iglhaut et al., 2019). Classical Structure-from-Motion (SfM) techniques have traditionally been used, relying on identifying and matching key points across multiple overlapping images to reconstruct 3D geometry. While effective, SfM can face challenges in capturing fine details or handling complex, occluded environments typical of natural habitats. Modern approaches, such as Neural Radiance Fields (NeRF), leverage deep learning to encode 3D scenes, enabling photorealistic reconstructions with a high level of detail, even in challenging scenarios (Ming et al., 2024). A newer technique, Gaussian Splatting, models scenes using Gaussian primitives to represent both geometry and appearance (Liu et al., 2024). This method is computationally efficient and excels in producing smooth, high-quality reconstructions of complex environments, including foliage or intricate terrain. As an alternative for real-time processing, Visual Simultaneous Localisation and Mapping (SLAM) techniques use camera data to enable drones to simultaneously map their surroundings and localize themselves (Barros et al., 2022), supporting autonomous flight and adaptive mission planning for wildlife observation. These methods are particularly suited for lightweight drones, providing detailed spatial data for applications such autonomous wildlife monitoring and environmental mapping without the need for additional LiDAR sensors.

## 3 WildDrone methodology

### 3.1 Scientific areas

The WildDrone research methodology and approach to address nature conservation challenges is based on developing novel drone- and computer-vision-driven technologies and to generate discoveries through ecological studies. The project holds high potential for a positive impact on nature conservation, and potential economic gains and growth from commercialization of innovations. WildDrone is founded on three major scientific thematic areas and relies on a cycle of iterative improvements where technological limitations are continuously balanced against domain requirements to achieve a synergy between drones, computer vision, and ecology. Forward interactions (new technology made available) and backward interactions (new requirements are set) will be pursued constantly in the project. They will be explicitly addressed during the two joint field trip hackathons in Kenya at Ol Pejeta Conservancy (OPC), where all DCs will jointly test their latest developments.

- Theme 1 will focus on innovative applications of drone technologies to ecological science and wildlife conservation. These DCs will produce ecological knowledge that is too costly and time-consuming to produce using conventional methods. They will start their research using consumer drones and state-of-the-art algorithms and will progressively integrate the advanced software and hardware developed by the other DCs. Additionally, they will assess the performance capabilities of low-cost, commercially available drones to determine which applications require more specialized and/or novel drone equipment.
- Theme 2 will innovate on drone design, operations, and control. These DCs will work together to develop a new generation of drones adapted to the needs of ecology and nature conservation. The technological development will encompass both highly versatile but costly designs suitable for long-range safety-critical operations, as well as simpler and less expensive designs suitable for end-users in low-income countries and resource-limited sectors.
- Theme 3 will develop computer vision techniques focusing on vision-based control, tracking, animal censuses, and individual characterization. These DCs will work together to produce software (onboard and desktop) specifically designed for animal conservation, with requirements and generalization abilities defined, studied, and validated by the ecologists in the consortium. They will explore the adequacy of citizen science to scale labels acquisition and individual identification, providing transferable tools scaling up research involving supervised computer vision for animal conservation and beyond.

Internally, technical requirements from Theme 1 on needs for capability developments in Theme 2 and 3 are documented as technical reports for review, as are requirements from Theme 3 on drone system capabilities. Nevertheless, in practice the hackathons are the main WildDrone method for ensuring the forward and backward interactions between the themes, as described in Section 3.2.

We will produce “science that matters” by developing technology in close collaboration with end-users. The challenges studied in Theme 1 are crucial and costly problems in wildlife conservation defined by our partners: wildlife movement and behavioural monitoring, human-wildlife conflict mitigation, and quantification of environmental parameters that affect animal physiology and behaviour. We will build a solid foundation for long-term, interdisciplinary European and African excellence and innovation in technology-assisted wildlife conservation. This will be achieved by facilitating cross-domain interaction through joint field work in ecological science and nature conservation practice; by sharing research infrastructures for field testing; and by disseminating the research and training outcomes and best practices of WildDrone in the doctoral schools of the partners and through public communication and events. We aim to foster long-term partnerships and collaboration mechanisms that will extend beyond the network’s timeframe.

### 3.2 Interdisciplinary collaboration

WildDrone brings together scientists from aerial robotics, computer vision and wildlife ecology in a true interdisciplinary collaboration. This is reflected in the joint use of themes to group DCs according to scientific areas, and in the use of collaboration clusters to support synergies between DCs across different scientific areas and in the training program in general. DCs will collaborate between themes by sharing technology and requirements with each other. The collaboration clusters indicate close interaction between specific DCs that act as catalysts for cross-theme interaction by directly supporting interdisciplinarity: DCs collaborate across themes to implement technological solutions relevant to nature conservation practice.

A key part of the WildDrone research approach is using *interdisciplinary hackathons* to encourage collaboration between different research areas. The project includes two hackathons, where all DCs work together in a real-world setting—specifically at OPC. Doctoral students from Theme 1 (conservation ecology) are familiar with fieldwork but need to explore the capabilities of the commercial-of-the-shelf technology and the new tools being developed in Themes 2 and 3. On the other hand, students from Themes 2 and 3 need to understand the needs of Theme 1 and test their prototypes in realistic conditions. This helps them see the limitations of their technology and find new ideas for their projects.

By bringing all the DCs together in the field, the hackathons not only promote teamwork but also provide opportunities to conduct meaningful scientific work collaboratively. The first hackathon takes place halfway through their PhDs and focuses on testing early prototypes. The second hackathon builds on these insights to experiment with more advanced, final versions of the technology. These two joint hackathons thus serve as the main WildDrone mechanism for ensuring that ecological needs identified in Theme 1 guide technological development in Themes 2 and 3. During the events, all DCs conduct joint field experiments, confronting Theme 2 and 3 students with the practical realities of fieldwork. Similarly, technical advancements from Themes 2 and 3 are tested experimentally in the field at these hackathons, creating a feedback loop that helps refine Theme 1’s research design for short-term field observations conducted using novel technology.

## 4 Automated wildlife tracking for conservation practice

WildDrone will explore innovative uses of drones for ecological conservation. This includes improving our understanding of animal behaviour through studies on resources use by migratory storks along the Western European Flyway (Section 4.2), improving monitoring methods for marine wildlife (Section 4.4) and managed populations of terrestrial megafauna (Section 4.5), and investigating the effects of prey presence on lion hunting and movement decisions (Section 4.1). The DCs will also focus on predicting and mitigating wildlife-related conflicts, including illegal poaching (Section 4.3) and problematic wildlife-livestock interactions in Kenya (Section 4.1), and disturbance of marine life by anthropogenic noise in coastal environments, such as the English Channel and the Wadden Sea (Section 4.4).

### 4.1 Fine-scale spatial behaviour of African lion (*Panthera leo*) in relation to wild and domestic prey

The spatial behaviour of apex predators, such as lions, is a crucial aspect of their ecology and has significant implications for the management and conservation of both these top predators and the ecosystems they inhabit (Kittle et al., 2016). Unique among felids for their social structure, lions have been extensively studied, especially for their group hunting behaviour and prey selection (Funston et al., 2001; Mbizah et al., 2020).

The presence and movement of lions within a landscape can significantly influence the distribution and abundance of their prey, thereby impacting predator-prey dynamics and overall ecosystem structure (Kittle et al., 2016; Loveridge et al., 2017; Mbizah et al., 2020). However, our understanding of the behavioural processes that guide lions in their spatial decisions, especially at finer scales, remains incomplete, which limits our ability to predict lion movement and mitigate lion-livestock conflicts (Abade et al., 2020; Davidson et al., 2012; Hebblewhite et al., 2005; Mbizah et al., 2020; Spong, 2002).

Recent studies have begun to shed light on these behaviours. For instance, lions have been observed to prioritize areas where prey is more accessible rather than abundant, suggesting a preference for spaces that facilitate ambush (Hopcraft et al., 2005; Mosser et al., 2009). Similarly, Valeix et al. (2010) found that lions tend to avoid repeatedly hunting within the same area, likely because prey in frequently targeted zones become more vigilant and enhance their defensive behaviour. These insights highlight the need for further research to develop a more comprehensive understanding of lion spatial behaviour and lion-prey interactions.

Drones have transformed wildlife conservation efforts in recent years, offering a host of advantages for researchers and conservationists (Barnas et al., 2020; Duporge et al., 2021; Mesquita et al., 2022; Schad and Fischer, 2023). Drones have become increasingly ubiquitous thanks to their methodological advantages (Aulia Rahman and Setiawan, 2020; Ivanova et al., 2022; Koger et al., 2023). They provide high spatial and temporal resolution data, are cost-effective, logistically convenient, and ensure the safety of researchers (Mesquita et al., 2022; Schad and Fischer, 2023; Beaver et al., 2020). This versatility has enabled their use in a wide range of wildlife-related activities, including detection, monitoring, and habitat assessment (Larsen et al., 2023; Schad and Fischer, 2023; Ivanova et al., 2022). For these activities, drones not only reduce data collection costs but can also result in less disruption to animals compared to traditional in-person surveys (Beaver et al., 2020; Mesquita et al., 2022; Hua et al., 2022). In this frame, the integration of aerial video-based observation with advanced machine learning-based image processing tools has emerged as a cutting-edge method for producing high-resolution movement datasets (Chen et al., 2023; Delplanque et al., 2022; Koger et al., 2023; Lenzi et al., 2023). These datasets are crucial for quantitative, multi-scale studies of wildlife behaviour, enabling researchers to conduct novel studies on how animals interact with their social, biotic, and abiotic environments (Koger et al., 2023). Such studies contribute to a more comprehensive understanding of the individual-level factors that drive broader ecological processes and patterns, as highlighted by recent research (Koger et al., 2023; Costa-Pereira et al., 2022). This synergy between technology and ecology holds the potential to transform our knowledge of wildlife behaviour and its implications for conservation (Koger et al., 2023; Pollock et al., 2022).

**FIGURE 1:**
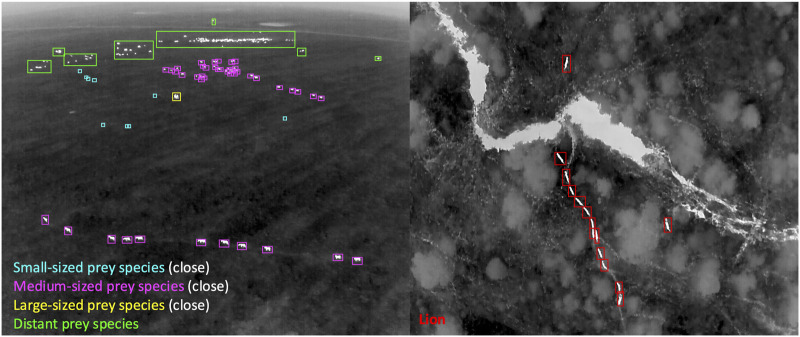
Thermal drone imagery collected during nighttime tracking operations with overlaid annotations indicating, on the left panel, prey species presence as small-sized prey, medium sized prey, large-sized and distant prey when too far to classify, and, on the right panel, lion’s movement.

In this doctoral project, we employ drone technology to generate new insights into lion spatial behaviour and fine-scale predator-prey interactions, with the potential to inform strategies for mitigating lion-livestock conflicts (Mogensen et al., 2011). The project consists of analyzing lion spatial behaviour by collecting data on lion activity, distribution, movement patterns, and interactions with prey to gain a comprehensive understanding of predator-prey dynamics. This approach accounts for habitat variability and prey encounters, and enables the exploration of nocturnal behaviour—an aspect traditionally difficult to study using conventional methods. Data collection is carried out during night time using a thermal camera–equipped drone, lions’ prides are localized with the help of VHF/GPS collars previously placed on dominant females. Once the target lion pride is found, the drone is used to 1) scan the surroundings to collect data on prey species presence, and 2) track the lion’s movement in “Nadir” view (camera pointing 90° perpendicular to the Earth’s surface). Then the visual data is processed through a detection model algorithms to count and identify general classes of animals as shown in Figure 1. Subsequently, detections of prey species are used to estimate their real-time abundance and distribution across the landscape traversed by lions, while lion detections provide tracking data on their movements. These data are then geo-referenced to interpolate nocturnal activity of the recorded animals in relation to habitat variability derived from available habitat maps.

### 4.2 Studying resource use by a long-distance migrant using drone technology

Animal movement is known to be shaped by the energy landscape, via variation in movement costs driven by variation in the physical environment (Wilson et al., 2012). Animals adapt their movement strategy following the temporal and spatial variations of the energy landscape to minimise the cost of transport (Shepard et al., 2013) while also maximising their access to resources, including food, mates, or social information (Williams and Safi, 2021). To limit energy expenditure during flight, large bird species depend mainly on soaring flight, whose costs are comparable to resting (Norberg, 1996; Duriez et al., 2013). During soaring, birds make use of vertical air movements known as thermal updrafts. These updrafts, or simply thermals, are formed by rising masses of warm air. Solar radiation heats the ground, which then heats the air above, causing it to rise (Ákos et al., 2010). When large birds encounter a thermal, they circle within and gain altitude. Then, after reaching a desired altitude or the top of the thermal, they glide forward until they reach another one. This soaring-gliding flight allows large birds to extract energy from the aerial environment (Williams H. J. et al., 2020) to reduce movement costs. Thus, thermals act as a valuable resource during migration.

The social landscape impacts the movement decisions of many species, which use the presence and movements of other birds to detect and estimate the quality of thermals (Williams et al., 2018; Sassi et al., 2024), including white storks (*Ciconia ciconia*) (Flack et al., 2018). This long-distance migrant travels in large flocks which can number up to thousands of individuals. Although this species has been the subject of numerous studies, quantifying the availability of social information during its migration remains challenging. Multi-individual tracking with GPS loggers can provide us with estimates of conspecific presence during migration (Brønnvik et al., 2024), but only from a limited number of individuals within a population. In the field, social information can be quantified with ground-based observation methods, such as carrying out censuses by scope. It is also possible to perform aerial observations with drones, which have the advantage of being deployable over areas that are not visible from the ground.

Yet, to understand how reliable social cues can be and to determine how different species should balance the use of personal and social information, it is essential to quantify the dynamics of thermals. Updraft availability varies considerably in space and time, depending on the underlying landscape and environmental factors like wind, cloud cover and solar radiation. Static landscape features can predict areas with suitable uplifts (Scacco et al., 2019), but uplifts are turbulent, dynamic, and often subject to wind drift and turbulence (Shepard et al., 2016). Large-scale patterns can be derived from models based on the energy landscape, but the fine-scale dynamics of the physical soaring environment are largely overlooked. This knowledge gap is largely due to the difficulty of obtaining these fine-scale data. In addition, past research on soaring flight has mainly been able to estimate updrafts at locations where the birds are, using the birds’ movements as an indicator of updrafts. But these indicators leave unmapped regions when there are no biologging observations. However, the rise of drone technology has provided novel tools capable not only of collecting data on these fine-scale air movements but also of doing so in previously inaccessible locations.

**FIGURE 2:**
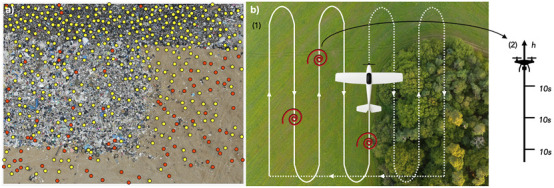
**(a)** Annotation of a drone-acquired picture at a landfill in Narbonne, France. Storks (yellow) and gulls (red) are labelled to help train an object detection model. **(b)** Representation of an experimental protocol to study the spatio-temporal dynamics of thermals. A fixed-wing is flown along a boustrophedon pattern over an area encompassing different habitats, collecting atmospheric data and imagery of the environment (1). When it detects an increase in vertical wind, indicating the potential presence of a thermal (red spiral), a quadcopter is deployed onsite: it climbs on a vertical profile, stopping regularly and measuring vertical wind for 10 s (2).

In our project, censuses are carried out at stopover sites along the migration path of white storks to study fluctuations in migratory numbers and compare methods for quantifying the availability of social information (Figure 2a). Drone technology is employed to measure atmospheric variables—including vertical uplift—and to capture environmental imagery (Figure 2b). These data enable the exploration of daily and seasonal dynamics within soaring environments encountered during migration. Furthermore, the study examines how habitat type, including anthropogenically altered landscapes, influences these dynamics. Understanding these patterns is essential for predicting migration costs across species and contributes to modelling the role of social information in migratory decision-making.

### 4.3 Differences between responses of prey to predators and humans

The non-consumptive and fear-driven effects of predation are important factors that influence ecological dynamics, shaping entire ecosystems through what is known as the ‘ecology of fear’ (Ripple et al., 2014). The perceived risk of predation can induce significant behavioural changes in prey species, such as increased vigilance, altered movement, and foraging patterns, which can ultimately affect their fitness and survival (Brown et al., 1999; Suraci et al., 2016). Although the anti-predator responses of wildlife to vocalisation cues of their natural predators have been studied extensively (Hettena et al., 2014), the fear induced by humans, and how it compares to those of natural predators, is a subject of ever-growing importance in the Anthropocene.

Humans are now recognised as a potent source of fear across the globe, capable of altering wildlife behaviour and survival in ways that may parallel or even exceed those of natural predators (Lasky and Bombaci, 2023). Since humans are responsible for a substantial portion of terrestrial vertebrate mortality (Hill et al., 2019), there is mounting evidence to support the hypothesis that many species fear the modern human “super predator” more than their natural predators (Crawford et al., 2022). Studies have shown that diverse species, from African elephants (McComb et al., 2014) to mesocarnivores (Clinchy et al., 2016), exhibit strong fear responses to human vocalisations. A recent large-scale study across the African savanna demonstrated that the mammalian community consistently showed a greater fear of human voices than of lions and other predators, reinforcing the idea that fear of human vocalisation is a pervasive phenomenon (Zanette et al., 2023). However, despite this growing body of evidence, there remains a critical need for research that directly and experimentally compares the behavioural responses of prey to both humans and non-human predators, combining vocal and visual cues, under controlled conditions.

This research aims to address this knowledge gap by systematically investigating how the anti-predator behaviour of free-ranging herbivores differs in response to disturbances caused by humans versus natural predators. We focus on two common prey species in the Kenyan savanna, plains zebra (*Equus quagga*) and impala (*Aepyceros melampus*), and their responses to cues from key predators—the lion (*Panthera leo*) and spotted hyena (*Crocuta crocuta*)—alongside human (*Homo sapiens*) cues. Our project moves beyond simple recordings of flight responses to quantify the variations in anti-predator behaviour, addressing whether prey animals differentiate between natural and anthropogenic threat types and adjust their anti-predator strategies accordingly.

To test these hypotheses, we have established a robust experimental framework to quantify the anti-predator responses of savanna herbivores. Randomized trials are conducted on independent groups of zebra and impala at artificial water throughs in Ol Pejeta Conservancy. The experimental design involves presenting standarized stimuli, including predator models (lioness and spotted hyena), a human mannequin, and pre-recorded vocalizations, using a custom-built, remotely-operated concealment system that reveals the stimulus on demand.

**FIGURE 3:**
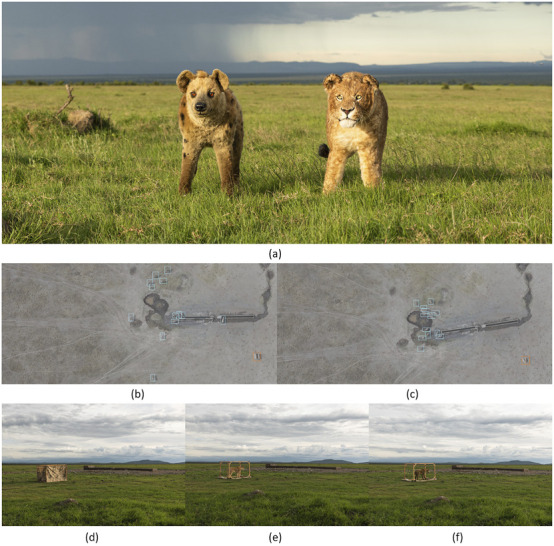
Experimental design and drone-based data collection for quantifying anti-predator behaviour. The figure illustrates the experimental stimuli and data processing workflow. **(a)** The two taxidermic predator models, a lioness and a spotted hyena, are shown together for scale. The middle panel shows the presentation method: **(b)** a group of 13 zebras approaches and drinks from a water trough, with individuals’ movements tracked using bounding boxes (teal) while the cage (orange) is in its pre-stimulus state; **(c)** the same group is tracked as they flee after the cage revealed the predator model. The spatiotemporal coordinates derived from these images are used to quantify key behavioural metrics, including individual escape trajectories, turning angles, and changes in inter-individual distances. The bottom two panels illustrate the data collection using drone imagery: **(d)** a remotely-operated concealment cage with its retractable sides up (pre-stimulus), and the cage revealing either **(e)** the lioness model or **(f)** the spotted hyena model.

We use UAV to collect high-resolution, nadir-perspective video data from 75 m above ground level (AGL) (Vacca et al., 2017). This allows us to unobtrusively capture the undisturbed approach of animal groups and their subsequent flight response following stimulus presentation. This expetimental workflow, from the stimuli to the drone-based data capture, is illustrated in Figure 3.

The high-resolution video data is being processed using computer vision and machine learning pipelines (Koger et al., 2023) to extract and quantify a suite of behavioural metrics. These include individual- and group-level vigilance, latency to flee, flight initiation distance, and escape trajectory characteristics.

Recognising that anti-predator responses are context-dependent, our analysis also incorporates how environmental and social factors shape these behaviours. We assess how habitat characteristics, such as vegetation density and structural complexity, influence vigilance and escape dynamics (Gigliotti et al., 2020; 2021; Fakan et al., 2023). Simultaneously, we analyze the social environment by accounting for group size, composition, and individual traits such (age and sex), which are known to affect risk perception and collective responses (Cresswell and Quinn, 2011; Beauchamp, 2019; Møller et al., 2016). This integrated approach provides a robust framework for understanding how prey species perceive and respond to threats from both natural predators and the human “super predator,” offering critical insights into the ecological consequences of human presence in wildlife habitats.

### 4.4 Tracking cetaceans in coastal areas

Monitoring large mammals in marine and terrestrial environments presents significant challenges, particularly in areas with low visibility or high human activity. Traditional methods often risk disturbing wildlife or require substantial resources, emphasizing the growing need for non-invasive remote technologies (Hodgson et al., 2016; Linchant et al., 2015). In marine environments for instance, detecting cetaceans during monitoring campaigns is often challenging, especially in expansive areas such as offshore wind farm sites, where traditional survey methods face significant limitations (Smith et al., 2020; Verfuss et al., 2019). Recent advances in both thermal sensors and UAV platforms have positioned drones equipped with thermal infrared (TIR) and RGB cameras as promising tools for developing innovative monitoring methods (Lonati et al., 2025; Meade et al., 2025; Seymour et al., 2017; Zhang et al., 2025). For instance, such systems can refine our understanding of human interference (Headland et al., 2021), help reduce disturbances to wildlife (Lonati et al., 2025), and enhance our ability to detect individuals (He et al., 2020; Seymour et al., 2017; Young et al., 2019). While both thermal signatures (Meade et al., 2025; Zitterbart et al., 2020) and indirect signs of presence (Cubaynes et al., 2019; Jewell et al., 2001; Tucker et al., 2024) have already proven valuable for detecting and tracking species, analyzing behaviour, and uncovering ecological patterns, combining these approaches holds particular promise (Churnside et al., 2009; Florko et al., 2021), especially in marine environments, where animals spend much of their time underwater, offering only limited windows of observation.

**FIGURE 4:**
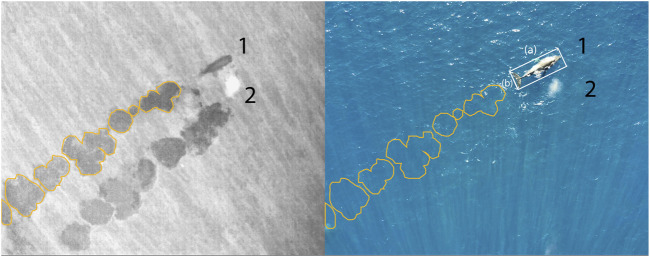
Synchronized drone-based imagery of two humpback whales (*Megaptera novaeangliae*) showing complementary thermal and visual perspectives of flukeprints and surface features. Left: thermal infrared (TIR) image, where two sets of flukeprints are clearly visible (whales numbered 1 and 2). Flukeprints for whale 1 are segmented in yellow, with the same segmentation overlaid on the corresponding RGB image (right) to indicate their positions, even when no flukeprints are visible. Right: RGB image, where only whale 1 is visible; whale 2 is not seen. A bounding box around whale 1 highlights measurements of **(a)** body length and **(b)** fluke span, illustrating how flukeprints can be used to estimate morphometrics and track movement dynamics of cetaceans.

Developing drone systems that dynamically minimize disturbance and utilise indirect cues represents a novel and impactful approach to wildlife monitoring. These systems enable high-quality, real-time observations while reducing the risk of interference with animal behaviour. Key research objectives include identifying environmental factors that influence the visibility of tracks and prints in drone imagery, and establishing standardized metrics for species detection, movement patterns, and behavioural assessments (Figure 4).

Building on the principle of minimizing disturbance, a non-invasive monitoring framework for terrestrial environments addresses whether existing general or species-specific guidelines for drone use around wildlife are sufficient or require refinement (Afridi et al., 2025). By integrating thermal imaging with AI-driven image analysis, our project explores species-specific responses to UAV presence under varying environmental conditions.

These efforts aim to establish practical methodologies for stakeholders in conservation and wildlife management, offering tools that enhance decision-making while minimizing direct interaction with animals. This approach significantly reduces the risk of disturbance and provides actionable insights for sustainable monitoring practices.

### 4.5 Improving wildlife monitoring with aerial photogrammetry: applications for marine and terrestrial large mammals

Drone-based wildlife monitoring methods can detect and count individuals of one or several species with speed and accuracy (Hodgson et al., 2018; Corcoran et al., 2021; Fettermann et al., 2022). Although counts can provide a valuable estimate of the state of a population, more detailed census data including age classes, sex, body condition, reproductive status, or other health markers are needed for a better evaluation of population structure and demographic trends (Christiansen et al., 2020; Rahman et al., 2023; Vermeulen et al., 2023). Collecting such information from minimally intrusive drone surveys using zoom photography and photogrammetry techniques would allow fine-scale monitoring of populations of marine mammals spread over large areas and facilitate the work of rangers and veterinarians managing and protecting large terrestrial animals in difficult field conditions.

**FIGURE 5:**
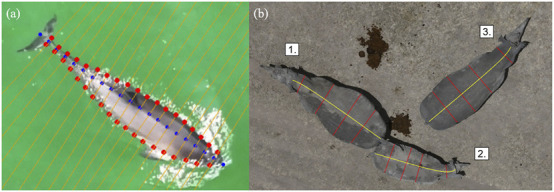
Nadir zoom images captured for the photogrammetric measurements of large mammals. **(a)** An adult harbour porpoise (*Phocoena phocoena*), with body width taken at intervals of 5% along the length of the animal. Total length, fluke width, and distance between blowhole and dorsal fin are measured separately. **(b)** A pregnant 8 year old female (1.), her 1.5-year-old female calf (2.), and a 5 year old male (3.) Southern white rhinoceroses (*Ceratotherium simum simum*). Yellow lines represent body length from the base of the tail to the top of the skull, following the spine curvature. The ratio between mother and calf length helps estimate the age of the calf. Red lines depict width at neck, shoulders, abdomen, and hips. We strive to determine which ratios between these measures can denote pregnancy, body condition, or sex.

Aerial photogrammetry has been used in various species of cetaceans to evaluate body condition (Christiansen et al., 2019), reproductive status (Cheney et al., 2022), energetics (Christiansen et al., 2016), and age class (Vivier et al., 2023). However, similar methods remain challenging to apply to harbor porpoises *(Phocoena phocoena)*, due to their small size, unpredictable swimming patterns, and the lack of obvious, individually distinctive markings (Elliser et al., 2022). Harbor porpoise populations in the Baltic and North Seas have undergone significant declines, with some regions like the Baltic Proper now critically endangered (Benke et al., 2014; Koschinski, 2001; Nachtsheim et al., 2021). Although monitoring methods such as acoustic surveys and stranding data provide valuable information, they have limitations in coverage and representativeness (Kyhn et al., 2012; IJsseldijk et al., 2020). This doctoral project calibrates and evaluates the precision of aerial photogrammetry methods by collecting data on harbor porpoises under human care—individuals that are regularly weighed and measured (Stepien et al., 2023) (Figure 5a). This controlled setting enables the development of recommendations for drone approach parameters and video processing techniques, with the aim of improving measurement accuracy in wildlife monitoring applications.

Photogrammetry methods would also be beneficial to complement the work of rangers monitoring rhinoceroses, by providing safe and lightweight tools that can produce standardized data on the animals under their protection (Inman and Leggett, 2022). For example, aerial measures could inform wildlife managers in real time about the age of calves or the pregnancy of females, or about the evolution of the body condition of animals during drought events, alerting authorities of the eventual need for food supplementation in fenced-in populations (Berger, 2012; Ewen et al., 2015) (Figure 5b). These non-intrusive techniques would advantageously complement traditional methods of observation where evaluations are subjective and require rangers to approach elusive, potentially aggressive animals on foot (Singh et al., 2020; Galliers et al., 2022).

### 4.6 Preliminary WildDrone results for automated wildlife tracking

In Theme 1, the early stages of the project have focused on designing and implementing field protocols to collect novel drone-based datasets for studying animal behavioural ecology and monitoring populations. Using drones to observe animals in natural conditions involves adaptation of conventional observational methods as well as flight protocols that allow safe operations in field environments and minimize disturbance to sensitive wildlife. Theme 1 DCs have successfully deployed drones in challenging terrestrial and marine environments to capture novel datasets on animal behaviour and movement. These include capturing synchronized thermal and RGB videos to track cetaceans using thermal signatures in the water (“flukeprints;” Figure 4); using thermal drones to survey distributions of ungulate prey at night and to track lions as they move through these prey landscapes (“prey scan and lion track”; Figure 1); using drones to perform photogrammetry measures of porpoises and rhinoceroses (Figure 5); and filming the behavioural responses of prey animals to predator models (Figure 3).

A critical component of behavioural research is understanding the impact of the observer on the subject animals’ welfare and behavioural patterns. In drone-based research, there is potential for negative impacts on wildlife due to the auditory and visual stimuli produced by the drone. Theme 1 and 2 DCs have conducted experiments in which target species (lions and zebras) are approached by drones flying at a range of altitudes and speeds, in order to understand the impact of drones and develop low-impact flight protocols for behavioural data collection. This work also connects strongly with research of one DC in Theme 2 that focuses on developing low-noise drone systems suitable for wildlife conservation applications (Afridi et al., 2025).

Finally, drones have great potential as a platform for observing and identifying individual animals for the purposes of population management, and may offer advantages over conventional ground-based methods. Theme 1 students have worked closely with conservation practitioners at Ol Pejeta Conservancy to understand current methods for monitoring lions and rhinoceroses and identify areas in which drones may be used to improve the accuracy, efficiency and safety of these operations. They have collected data that they will use to compare ground-to drone-based methods and develop tools to reduce the human burden of wildlife monitoring and data interpretation.

## 5 Safe and flexible drone operations

WildDrone advances drone design, operations, and control through several key innovations. These include the pre-mission modification of drone noise profiles to minimize disturbance to wildlife (Section 5.1); the acquisition of accurate, real-time animal geolocation data using off-the-shelf UAVs (Section 5.2); integration with UTM systems to BVLOS conservation missions across Europe and Africa (Section 5.3); and the development of planning systems for coordinated multi-drone data capture (Section 5.4). These technological advancements are designed to enhance ecological monitoring while reducing interference with animal behaviour.

### 5.1 Drone noise profile optimisation for its impact on animal behaviour

Deploying drones for ecological monitoring has opened up new possibilities in wildlife research. They offer efficient, adaptable, and low-impact ways to gather data, enabling mapping of habitats in high detail, observe animal behaviours, and survey populations across wide or remote areas (Elmore et al., 2023). But as drones become more common in conservation work, it is crucial that we also address the unintended consequences, especially drone noise disturbance, and find effective ways to minimize those impacts (Afridi et al., 2025).

Noise generated by drone rotors constitutes an immediate source of disturbance. A substantial body of empirical research indicates that such acoustic emissions can trigger stress responses, alter animal behaviour, and ultimately compromise animal welfare (Mesquita et al., 2022; Scobie and Hugenholtz, 2016). These disturbances also risk introducing biases into scientific data, as animals may flee, freeze, or otherwise change their natural activity in response to drone presence (Ditmer et al., 2015; Shannon et al., 2016). Auditory sensitivity is in general unique to each species: plain zebras (*Equus quagga*), for example, are highly attuned to low-frequency sounds and may react strongly; giraffes (*Giraffa camelopardalis*), more visual, respond very differently to the same acoustic stimuli. This interspecific variation highlights the need for a refined, species-specific understanding of how drone noise affects wildlife.

Despite increased concern on the issue, current methods for assessing drone noise impacts remain fragmented. *In situ* noise measurements are complicated by vegetation absorption, wind turbulence, reflections of sound through the varied terrain, and variations in atmospheric conditions, all contributing to altering the spectral and spatial characteristics of sound. Thereby, field conditions are quite different from laboratory conditions under which most acoustic data are gathered (Macke et al., 2024). Numerous studies focus either on documenting the presence of drone noise under controlled conditions (Rümmler et al., 2016) or observing wild animal reactions without the accompanying acoustic data (Duporge et al., 2021). Only a handful integrate these two approaches within a unified framework. This heterogeneity makes it difficult to compare results between studies, and develop pragmatic recommendations to minimize drone-induced disturbance.

Our project addresses limitations in wildlife-compatible drone design by integrating rigorous acoustic characterization with behavioural assessments of animal responses to drone exposure. The objective is to develop drone configurations that are both acoustically transparent and minimally disruptive to wildlife. This dual consideration is essential: while reducing acoustic emissions can mitigate disturbance, fully silent drones may raise ethical concerns related to surveillance and misuse. To support this goal, our project includes detailed acoustic testing of various drone designs under differing flight and environmental conditions. These tests measure objective sound features such as frequency spectra, harmonic content, loudness, and directionality. Acoustic signatures are evaluated against available wildlife audiograms to identify frequencies and intensities likely to elicit behavioural responses.

In parallel, the project develops bespoke noise mitigation strategies aimed at reshaping the acoustic footprint of drones without compromising operational performance. These strategies include modifications to propeller dimensions, blade number and shape, and the design of trailing edges and tips, as well as the potential use of sound-absorbing or deflecting materials. Behavioural experiments with captive animals are conducted to assess detection thresholds and responses to different drone types and sound profiles. By quantifying vigilance, evasion, and habituation behaviours under controlled conditions, the study contributes to predictive models of species-specific reactions to drone noise.

Overall, this research establishes a comprehensive framework for the design and deployment of wildlife-compatible drone systems by integrating acoustic engineering with ecological and ethological inquiry. The outcomes are expected to elevate ethical standards in drone-based field studies and enhance the reliability of ecological data collected via unmanned aerial platforms.

### 5.2 Accurate ground animal geolocalisation

The capacity to measure absolute and relative positions of animals in their environment is fundamental to understanding behaviour, ecological dynamics, and conservation needs (Koger et al., 2023; Costa-Pereira et al., 2022). Absolute positions inform analyses of space use, environmental interactions, and community-level effects. Whereas relative positions inform social dynamics, group cohesion, and collective movement such as foraging, predator avoidance and movement decision (Koger et al., 2023; Westley et al., 2018; Duporge et al., 2024; Kavwele et al., 2024).

**FIGURE 6:**
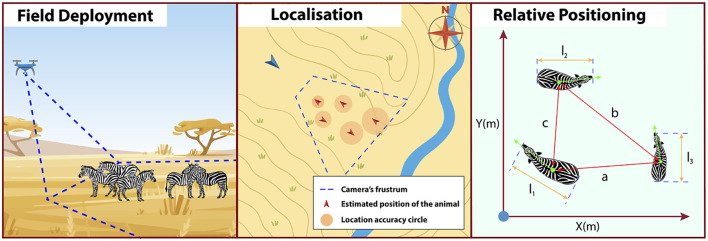
Deployment of UAV-based geolocalisation measurement (left panel), including positioning uncertainty (middle panel) and relative positioning (right panel) (Meier et al., 2025).

Conventionally, radio collars or biologging tags are used to locate and track individuals. These methods are, however, invasive, usually limited to single individuals, and often require the animals’ capture and handling (Duporge et al., 2024; Mesquita et al., 2023; Kavwele et al., 2024; Koger et al., 2023). In contrast, UAVs have the potential to provide non-invasive, high-resolution, real-time localisation of multiple individuals (Figure 6), improving behavioural and ecological observations also in remote areas (Duporge et al., 2024; Schad and Fischer, 2023; Koger et al., 2023; Mayer et al., 2024; Wirsing et al., 2022).

UAV-based wildlife geolocalisation can be achieved in real-time using well-known algorithms such as monoplotting (Meier et al., 2024) in combination with machine vision object detection and tracking algorithms (see Section 6). Our project explores and enhances the capacity of UAVs to monitor wildlife within their environmental and social contexts, aiming to establish field-tested and validated measurement methods. A central objective is to assess the geolocation accuracy and practical suitability of commercially available UAVs, which are already widely adopted within the biological research community. Building on this characterization, the project implements a time-filter-based algorithm to improve geolocation precision without relying on digital elevation models—a key limitation of current approaches. Additionally, geolocation data are used to inform a path-planning system designed to minimize localization uncertainty. This methodology also holds promise for mitigating double-counting in large-scale surveys involving moving animals, by enabling the prediction of individual trajectories.

### 5.3 Safe BVLOS operation of drones for nature conservation

Achieving safe BVLOS operations in unstructured and wildlife-rich environments remains a fundamental challenge for the widespread deployment of drones in nature conservation. Building on joint field deployments in Kenya, our project explores how regulatory frameworks such as the Specific Operations Risk Assessment (SORA) can be adapted and operationalized for missions in low-infrastructure settings. Recent work has demonstrated how real-world deployments can both inform and validate BVLOS-specific risk models through a combination of tailored tools, operational procedures, and field-based testing (Maalouf et al., 2025).

Key components of this work include the design and implementation of lightweight digital infrastructure to support daily mission planning, risk mitigation, and team coordination. A prototype UTM-lite system, referred to as WildOps, has been developed to enable the logging, visualisation, and coordination of simultaneous drone operations over protected areas. Coupled with this system, a modular checklist generator, WildProcedures, has been created to automate and adapt standard operating procedures to the constraints and objectives of each mission. These tools have been field-tested during large-scale joint operations at Ol Pejeta Conservancy in Kenya, where conservation-focused BVLOS missions were carried out across multiple days and teams under live conditions.

The field deployments provided insight into key challenges associated with BVLOS operations in dynamic airspace with unpredictable human and wildlife activity. These include difficulties in defining appropriate contingency areas, coordinating parallel operations in the absence of cellular coverage, and ensuring airspace deconfliction when formal UTM services are unavailable. The iterative deployment of WildOps and WildProcedures enabled more structured team coordination, clearer task allocation, and improved transparency in flight planning, highlighting the role of context-specific tooling for safe and scalable BVLOS missions.

To complement these operational tools, a broader review of airspace situational awareness strategies has been conducted to examine how drones can perceive and respond to aerial threats in the absence of national UTM infrastructure. This review synthesises existing detection methods, including ADS-B, radio frequency monitoring, acoustic sensing, and computer vision, and assessed their applicability for integration into conservation drone systems (Maalouf et al., 2024). Based on this analysis, a conceptual framework has been proposed for combining local sensing with cooperative field inputs to maintain situational awareness during BVLOS flights in remote regions.

### 5.4 Automated planning of safe, multi-drone nature conservation missions

**FIGURE 7:**
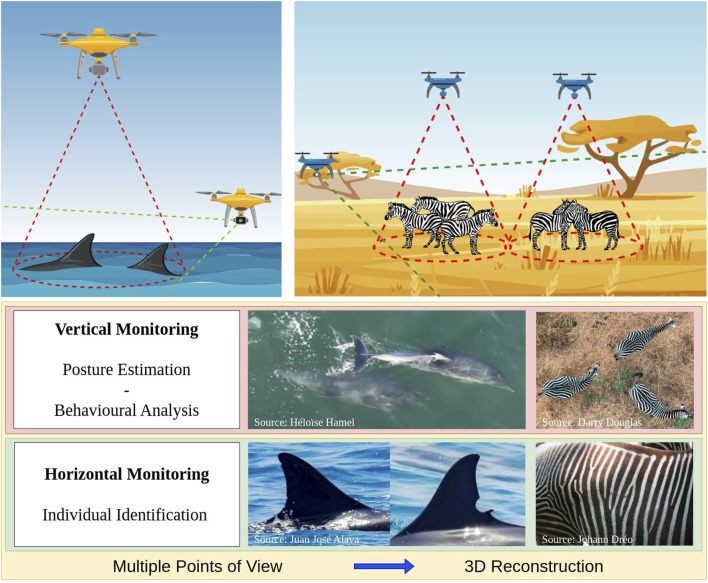
Motivation behind multi-perspective monitoring (Rolland et al., 2025c). Reproduced from Advancing Wildlife Monitoring in Gregarious Species with Drone Swarms, Rolland et al., Springer Nature, 2025, with permission. Not covered by the article’s CC-BY license.

Deploying multiple drones for simultaneous data collection significantly enhances the scope and efficiency of conservation ecology campaigns. Drone swarms have already proven effective for mapping tasks by enabling coordinated operations over large areas and reducing overall mission time (Bähnemann et al., 2019; Grøntved et al., 2023). However, deploying multiple drones for wildlife monitoring remains challenging. Biologists still rely largely on manually flown single-drone missions to gather biologically meaningful data (Koger et al., 2023). This approach has inherent limitations, including a restricted field of view and the limited autonomy of a single drone (Kline et al., 2025a). To our knowledge, only a few studies have demonstrated field-tested, autonomous multi-drone systems for wildlife conservation missions. While previous studies have employed multiple drones, they were generally manually operated (Shukla et al., 2024b). In our project, we focus on the use of multi-drone systems for multi-perspective monitoring. Leveraging multiple viewpoints allows the collection of richer datasets by combining complementary visual information. This enables individual identification, posture analysis, and group-level behavioural interpretation, as illustrated in Figure 7 (Inoue et al., 2019; Maeda et al., 2021).

Our project deploys drone swarms capable of multi-perspective data collection on wildlife species in natural habitats, supported by a flexible deployment strategy that can accommodate diverse species and mission objectives. The system is designed to be non-intrusive and accessible to users with no prior drone expertise. Tracking strategies are informed by biological data collection priorities and implemented to operate with minimal user intervention. The approach is validated through field experiments using real drones, demonstrating both the practical relevance of the method and the feasibility of simultaneous multi-drone deployments for effective wildlife monitoring (Rolland et al., 2025c).

### 5.5 Preliminary WildDrone results for safe and flexible drone operations

The challenge of selecting robust and cost-effective platforms for scientific drone operations was addressed through the design of WildBridge, an open-source system that enables lightweight multi-drone control and telemetry on entry-level DJI platforms (Rolland et al., 2025a). Built on DJI’s Mobile SDK, WildBridge provides standard network interfaces for telemetry, control, and video streaming, enabling integration with research frameworks such as ROS 2 and Python. The system was successfully applied across multiple research scenarios presented below, demonstrating reliable performance under realistic conditions while making reproducible drone research more accessible to non-experts.

Extending this framework, Meier et al. (2024), Meier et al. (2025) systematically characterised the geolocalisation and relative positioning errors arising when using the studied monoplotting algorithm, presenting a theoretical error model validated via simulation, controlled outdoor experiments in Bristol (UK), and live animal tracking at Ol Pejeta Conservancy (Kenya). This work also quantified the contributions of key error sources (e.g., GNSS, barometric altitude, camera attitude, object detection) to overall localisation and size measurement accuracy. Based on these findings, deployment recommendations are provided to achieve a desired geolocation precision.

**FIGURE 8:**
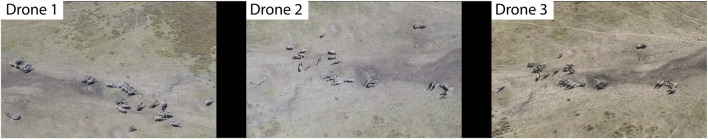
Example of multi-perspective imagery collected (Rolland et al., 2025b). Figure adapted from Kline et al., 2025, licensed under CC BY 4.0.

The problem of effective multi-perspective data collection with a drone swarm was formalised using the novel concept of Surfaces of Interest (SIs), which represent biologically relevant regions of the animal body to be observed. This formalisation allows the definition of an objective function that quantifies monitoring quality by considering factors such as viewpoint angle, image resolution, and animal disturbance, thereby ensuring that the data collected by the drones captures the parts of the animals needed to address the biological question while minimising disturbance (Rolland et al., 2024). The approach was first validated in simulation using a 3D environment and real animal spatial distributions. Drone configurations were optimised based on the objective function using particle swarm optimisation (Rolland et al., 2024). Then, we developed a working prototype of the system using commercial off-the-shelf drones integrated into a centralised control architecture based on ROS2 Rolland et al. (2025a). Finally, the system was successfully deployed and tested in the field during a 6-day campaign at Ol Pejeta Conservancy (Kenya), where twelve missions were conducted (Figure 8). These trials demonstrated the feasibility of our approach for real-time multi-perspective data collection under challenging real-world conditions. However, the results also revealed points of improvements such as enhancing the drone swarm’s responsiveness to dynamic animal movement and reducing reliance on manual animal detection to achieve full autonomy (Rolland et al., 2025b).

Complementary efforts within the consortium have also focused on quantifying and mitigating the impact of UAV disturbances on wildlife. Afridi et al. (2025), Afridi et al. (2024) synthesised current knowledge on behavioural and physiological responses of animals to drone exposure, identifying key disturbance pathways and highlighting the need for integrated acoustic–behavioural field validation. Building on this foundation, ongoing experiments at Ol Pejeta Conservancy (Kenya) are assessing species-specific response thresholds during single- and dual-drone missions. These preliminary findings are being used to inform the design of low-disturbance, wildlife-compatible drone systems by linking acoustic characterisation, behavioural sensitivity, and propeller-level aerodynamic optimisation.

Complementing these developments, recent field trials conducted at Ol Pejeta Conservancy in Kenya demonstrated the feasibility of conducting safe, multi-team BVLOS operations in support of wildlife research (Maalouf et al., 2025). Using the SORA 2.5 methodology as a planning framework, missions were performed across mixed terrain and active wildlife zones, supported by locally coordinated authorisations from the Kenya Civil Aviation Authorities and the Kenyan Air Force. Two lightweight digital tools, WildOps and WildProcedures, were deployed to facilitate tactical airspace coordination and automate checklist generation, respectively. These systems improved procedural consistency and reduced coordination workload, enabling teams to maintain safe separation without national UTM infrastructure. Preliminary analyses highlight that structured planning and software-enabled execution can substantially increase operational scalability while preserving safety and regulatory alignment, providing a reusable model for future conservation missions.

## 6 Effective computer vision for conservation

WildDrone develops computer vision techniques focusing on vision-based control, tracking, animal censuses and individual characterization: new techniques based on deep learning for large-scale animal censuses, allowing herds and individuals detection across nature reserves (Section 6.1); reconstruction of the 3D environments from drone flights, providing context information essential for behavioural ecologists (Section 6.2); single- and multi-animal (herd) flight tracking, as a essential tool for planning flights able to follow animals in the wild and acquire the most useful images across complex backgrounds and with sudden changes of trajectories (Section 6.3); and techniques for individual animal recognition (based on fur or physiological features), posture estimation and monitoring of the health of individuals of endangered species (Section 6.4). These four independently usable components answer critical needs in modern animal conservation: they develop novel capabilities that can be used together with the drone systems developed in Theme 2 to address the ecological scientific problems identified in Theme 1.

### 6.1 New census approaches robust to spatial and temporal variations

Frequent and consistent monitoring of animal populations is a key requirement for successful wildlife management and is particularly important when dealing with endangered species. In light of the safety risks and the limited scalability of conventional monitoring approaches (e.g., manned aircrafts and camera traps), there is growing interest in exploring the potential offered by drones for these purposes (Linchant et al., 2015; Chrétien et al., 2015; Nicolas et al., 2017; De Kock et al., 2022). Therein, convolutional neural network (CNN) based approaches are rapidly gaining popularity for detecting and counting animals in the imagery recorded by UAVs (Maire et al., 2015; Kellenberger et al., 2018; Peng et al., 2020; Dujon et al., 2021; Rančić et al., 2023).

While CNNs hold the promise of high detection accuracy, which can surpass that of humans (Torney et al., 2019; Wosner et al., 2021; Fan et al., 2023), this potential is contingent on the volume of labelled data available during training (Alzubaidi et al., 2021). Moreover, for drone-based wildlife detection this training data must not only be abundant, but also include representative samples for the different environmental conditions in which the animals of interest can be found. More specifically, CNNs can fail to maintain performance when applied to images from different habitats, or from different parts of a single habitat, that contain unseen types of soil and vegetation not included in their training (Kellenberger et al., 2019).

A further complication is posed by the fact that CNNs suffer from *catastrophic forgetting*, which means that they struggle to learn incrementally without forgetting previously acquired knowledge (Hadsell et al., 2020). In combination with the aforementioned variability of habitats, this means that obtaining a model that is suitable for robust and long-term monitoring of wildlife across landscapes would require a large training dataset containing examples of every animal class under all possible environmental conditions to be available at once. This is, of course, extremely challenging in terms of data logistics; especially due to the sensitive nature of conservation data (Cooke et al., 2017), which makes data sharing undesirable.

Our project addresses the limitations of current object detection technologies by exploring strategies to reduce the labelling cost associated with state-of-the-art models. It combines these efforts with approaches from federated learning and domain adaptation to develop a framework capable of progressively and efficiently learning to detect animals in previously unseen environments. The framework is designed to preserve existing knowledge and operate without the need for direct sharing of sensitive data or access to a comprehensive training dataset.

### 6.2 Reconstructing natural habitats from multimodal drone measurements

Reconstructing natural habitats is crucial for accurately monitoring and understanding ecosystems, forming a key component of autonomous drone-based wildlife observation systems that enable data-driven conservation efforts to protect biodiversity and mitigate environmental challenges (Haalck et al., 2020). Drones are particularly suited to generate 3D reconstructions due to their ability to capture high-resolution imagery from multiple angles, enabling detailed and flexible mapping of complex and inaccessible environments. Moreover, the mesoscale imagery provided by drones offers high-resolution, context-rich insights into habitat structure and wildlife interactions at a scale critical for effective conservation planning. This imagery can be integrated with high-accuracy georeferencing data from GPS and RTK systems, as well as multispectral sensor data, enabling detailed forest health monitoring. This multimodal dataset offers powerful opportunities for advanced habitat analysis and ecological assessment.

In spite of advancements in computer vision and multi-view geometry, there remain numerous challenges when working in unconstrained wildlife environments. First, UAV flights are sensitive to environmental factors like occlusions (Xu et al., 2024). For example, drones often record images in a nadir setting, capturing top-down views with the camera oriented directly downward. In such cases, natural elements like dense tree canopies, overhanging branches, or cliffs can obstruct parts of the scene, resulting in void regions that lack dense 3D point coverage. Secondly, despite the remarkable results of algorithms like SfM and SLAM, most approaches operate under the assumption that the observed environments are static (Saputra et al., 2018). However, when data is captured by drones in natural environments, dynamic elements such as wind-induced trees motion or animals moving through the scene introduce temporal and spatial inconsistencies between images. These inconsistencies aggravate the reconstruction process and reduce the accuracy of the resultant 3D point cloud. This challenge is particularly difficult for complex geometries since no geometric priors or motion models can be applied to capture the dynamics of these objects (Risse et al., 2018). Finally, textureless surfaces fail to provide sufficient feature points required for photogrammetric reconstructions, which depend on distinct patterns to accurately compute the 3D surface structure (Hafeez et al., 2020). This challenge is particularly pronounced in natural environments with uniform regions such as deserts or bodies of water. Researchers often use additional sensors (e.g., LiDAR) or algorithms to enhance textureless regions.

To address current limitations in habitat reconstruction, this doctoral project develops an innovative approach aimed at enhancing both the quality and accuracy of environmental models. Optimal imaging conditions and flight paths are assessed to reduce occlusions, thereby improving visual coverage of complex terrain. In parallel, multimodal data fusion techniques are employed to enhance reconstruction fidelity in dynamic environments. A combination of SfM and advanced machine learning methods, such as semantic segmentation, is implemented to address challenges posed by dynamic objects and textureless surfaces. These algorithms contribute directly to autonomous habitat mapping and wildlife monitoring, facilitating the generation of more accurate and comprehensive representations of natural habitats, supporting ecological research and conservation planning.

### 6.3 Adaptive tracking for detection and identification

Biogeography, population ecology, and behavioural research all critically rely on detecting species, individuals, behaviours, and morphological traits by phenotypic appearance, that is on performing animal biometrics as defined in Kühl and Burghardt (2013). However, reliable *in-situ* animal biometrics that go beyond species recognition (e.g., Sakib and Burghardt, 2020; Brookes et al., 2024; Kholiavchenko et al., 2025) require reactive navigational decision making to position the drone relative to moving animals such that biometric measurements can be taken correctly. This could be animal identification by coat pattern requiring visual access to the animals’ flank (Stennett et al., 2022), and behavioural fingerprinting or social recording of individuals within groups where a herd-view must be maintained. Deep detection at high resolutions resolves the underlying in-frame localization task by utilising species-specific appearance information acquired during network training whilst tracking approaches provide spatial-temporal localization and identity continuity at moments when detection within a region cannot be afforded due to resource constraints. Yet, as Xiao et al. (2023) highlight, integrating such computer vision outputs directly into control mechanisms of drones remains a significant challenge due to a plethora of technical and operational limitations, such as handling noisy or incomplete data, ensuring robustness in dynamic environments, as well as operating within the constraints of onboard computational resources.

In autonomous settings, facilitating effective animal-aware navigation requires near real-time multi-animal tracking onboard—that is to avoid the need for remote processing hampered by extended latency, limited bandwidth, or high cost in case of satellite links. Existing trackers (Karaev et al., 2023; Doersch et al., 2023; Tumanyan et al., 2024) are powerful but relatively slow for robotic navigation. Therefore, ultra-fast video-based dynamic tracking approaches are researched by us in order to deliver speed at high frame resolutions and accuracy, whilst processing live video streams on-device for close-to-instant navigational inference and mission-based decision making. In our project, we work towards interlinking and extending state-of-the-art deep object detectors and trackers running on an onboard GPU with ultra-fast tracking methods under mission-specific policies. Our goal is to provide maximum tracking accuracy and robustness with minimal computation to enable autonomous animal-aware navigation. These techniques have to be powered by onboard GPU hardware, such as the NVIDIA Jetson platform (Scalcon et al., 2024), in order to deliver the required computational footprint.

To this end, in our project, we apply dynamic tracking to a variety of critical tasks and missions, including: tracking of individual animals within a herd, tracking an entire herd with their individuals and capturing social behaviours therein, and (re-)identifying individual animals of interest for measuring longitudinal indicator biometrics or allowing for conservational intervention. In the context of wildlife conservation, this approach is particularly valuable for biologists working in large-scale or high-risk environments, where traditional methods of monitoring and intervention may be too time-consuming, costly, or invasive. By using drones equipped with advanced computer vision algorithms, conservationists can monitor large areas in real-time as discussed in (Iglay et al., 2024), allowing them to quickly identify changes in animal behaviour, detect distress signals, and pinpoint animals in need of immediate rescue. This capability is especially important in regions facing poaching threats, habitat destruction, or environmental changes, as it enables more effective and timely responses to crises. Ultimately, animal-aware tracking and navigation technology will help safeguard endangered species beyond the horizon by providing accurate, non-intrusive monitoring that can support decision-making and resource allocation in conservation efforts.

### 6.4 Detecting posture, metrics and biometrics of animals from drone data

Monitoring the health and behaviour of free-roaming wildlife species in their natural environment is crucial for conservation and ecological research. Animal biometrics, shape, and posture provide information temporally interpretable as health condition and behaviour of animals in complex broad-scale surveys (Koger et al., 2023; Tuia et al., 2022). Studies have shown that animal traits evolve in response to environmental changes (Cui et al., 2020) and can be critical in 1) studying their response to global crises (Ivanova et al., 2022) such as climate change or excessive land use, and 2) deciphering the response of wildlife to conservation efforts such as translocation or reintroduction of species to new areas (Petso and Jamisola, 2023).

Drones offer new opportunities for performing scalable, repeatable, and non-invasive analysis of animal biometrics, shape, and posture. They can help capture data from multiple opportunistic perspectives with high flexibility (when compared to camera traps) with possibilities for closer approach (when compared to remote sensing), making them a promising platform for studying individual characteristics with scope for computer vision based automation in processes such as shape extraction and posture estimation. Oblique views are rich in visual cues and allow to recover the shape, movement, and identifiable coat patterns of the animals within their ecological habitat (Shero et al., 2021). There exist several challenges, such as lack of datasets and ground truth, ill-sensor positioning, occlusion by vegetation and tree canopies, continuously moving animals, and diversity of shapes and appearances in different species, introducing several setbacks to automation of individual characteristics analysis (Shukla et al., 2024a; Xu et al., 2023). It remains almost impossible to place targets or have ground control points in the wild close to animals, and aerial non-nadiral imagery additionally suffers from ground distortions that make automated studies of morphometric nature complicated, prompting research in this avenue.

The main purpose of our project is to create novel methods for reliable, scalable, and economical individual characteristics estimation through utilization of computer vision techniques such as photogrammetry and pose estimation on drone-based data. The second goal is to enable 3D perception for drone cameras, supporting autonomous re-routing to achieve safe and efficient scene surveys. This capability is motivated by studies showing that autonomous systems often yield higher amounts of usable data during fieldwork for animal behaviour observations (Rolland et al., 2025c; Saffre et al., 2024). Technological developments for safe and effective focal monitoring operations in remote, unstructured environments will improve our understanding of complex wildlife systems to better frame conservation policies.

### 6.5 Preliminary WildDrone results for effective computer vision

The research directions highlighted in Sections 6.1–6.4 sketch a unified vision-based monitoring system that covers the whole chain, from smart data acquisition through tracking, to animal counting and biometrics estimation, alongside the monitoring of the environment. Moving toward this vision, we present here the initial results of the team.

**FIGURE 9:**
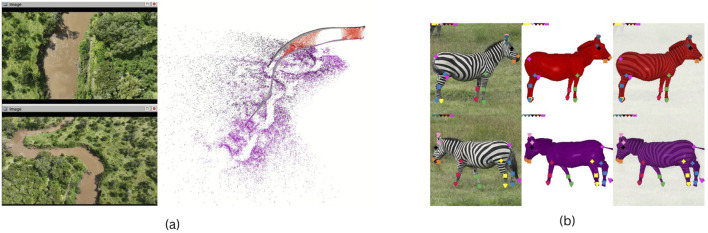
**(a)** Real-time extraction of drone camera trajectories from collaborative agents flying together in the same region. From Shukla et al. (2024b) **(b)** 3D shape and pose fitting results on zebras from multiple drone frames using drone camera trajectory and GNSS from flight logs: the results show automatically extracted 3D joints and 3D shape without/with superimposed original image. From Shukla et al. (2024a).

**FIGURE 10:**
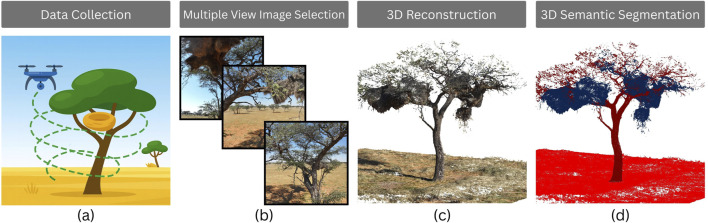
Workflow of tree and habitat (here nest) analysis using drone-based photogrammetry: **(a)** Data collection with UAV flights around the tree (nest is given in yellow), **(b)** multiple view image selection, **(c)** 3D reconstruction of the tree, and **(d)** 3D semantic segmentation of tree components (nest points are shown in blue).

Figure 9a shows results from our evaluation of real-time drone positioning and camera trajectories of multiple drone agents that are simultaneously acquiring data in outdoor settings (Shukla et al., 2024b). Images on the left of the panel indicate camera perspective of each agent at a particular time during the flight, and on the right are camera trajectories of both the agents in the same world reference along with sparse 3D reconstruction (red lines indicate the location matches in key frames), obtained using CCM-SLAM (Schmuck and Chli, 2019). Such a reconstruction serves as the starting point for detailed habitat reconstruction. The level of detail in habitat reconstruction can vary depending on the intended application. For large-scale surveys, coarse reconstructions such as vegetation maps, plant distributions, or digital surface and height models may already provide sufficient ecological information. However, since individual plants (and particularly trees) serve as essential habitats for many animal species, numerous applications demand more detailed three-dimensional representations. Achieving such reconstructions is challenging due to the structural complexity and fine geometrical features of vegetation. In a first study, we investigated the use of state-of-the-art 3D reconstruction techniques to obtain highly detailed models of individual plants, which were subsequently used to derive semantic graph representations for assessing physiological traits (Molina Catricheo et al., 2024). Extending these algorithms with drone-based imaging offers a powerful framework for autonomous wildlife and habitat analysis. A representative example of such an in-plant habitat is the nest of the sociable weaver. Analyzing these complex communal nests through 3D reconstructions provides crucial insights into habitat usage, colony structure, and nesting behaviour, which are key indicators of ecosystem health and species interactions in arid environments. Figure 10 illustrates how drone imagery can be employed to generate accurate 3D reconstructions of trees and associated nests. Additional 3D semantic segmentation enables the extraction of detailed quantitative information about the tree, the nest, and their surrounding environment.

**FIGURE 11:**
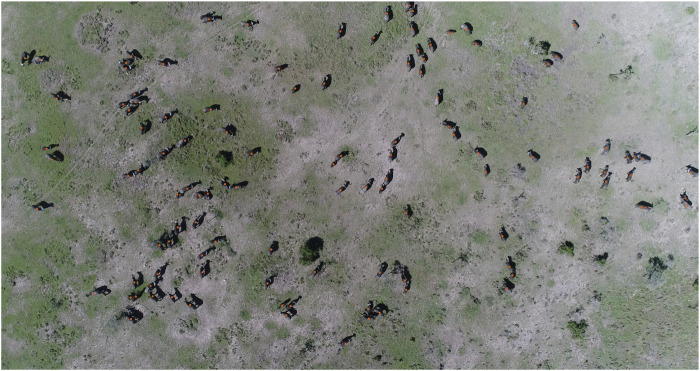
Visual examples of animal detections using the POLO model (May et al., 2024) on nadir drone images of an African buffalo herd. Predicted locations of animals are marked with dots, whose colour encodes the predicted class. Here, orange = buffalo.

Sequences of images taken on flight are used to develop new machine learning algorithms for fast automatic censuses, with a main focus on reducing the annotation load. The Point-based, multi-class animal detection algorithm [POLO, May et al. (2024)] allows detection of single animals using only point annotations, which are far more effective [by a factor 7, Ge et al. (2022)] in terms of annotation time with respect to the bounding boxes typically required by common object detectors such as the YOLO algorithm (Jocher et al., 2023), while providing comparable accuracy (May et al., 2025). POLO is based on the YOLOv8 architecture, to which several modifications to ensure point-compatibility were made, including architectural changes, replacement of the original loss function, and the definition of a new evaluation metric to measure detection accuracy. Figure 11 shows preliminary detection results on images taken in the Mpala and Ol Pejeta conservancies in Laikipia, Kenya (Koger et al., 2023). Orange points are the algorithm’s detections.

**FIGURE 12:**
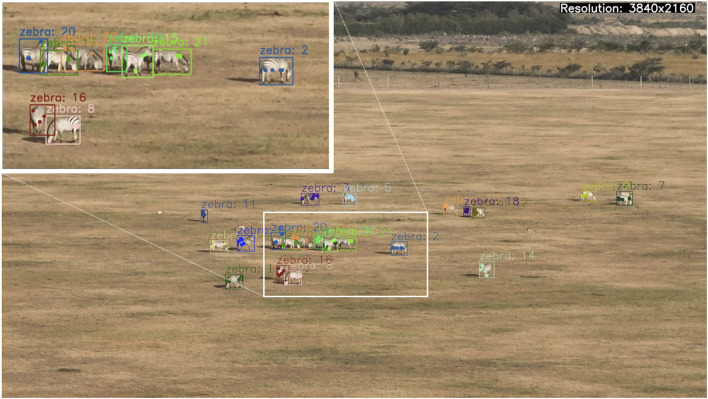
Example video frame where multi-animal tracking is applied to WildDrone data filmed in Kenya. We target near real-time speeds well above 10fps on 4K video in order to feed dynamic positional information of animal presence and identity towards decision making for live navigation. Zoomed-in region in the top left shows tracking of animals in dense groups with partial occlusions as often encountered in herd settings.

Drones acquire images in video mode in our experiments, and we use these videos for several purposes. First, videos circling around the animals are being studied for single animal pose and joint estimation: in Figure 9b, we show 3D shape reconstruction and joints estimation of single zebras from a video acquired in Ol Pejeta, Kenya (Shukla et al., 2024a). The video is processed by a series of algorithms in cascade, including object detection to retrieve the animals (He et al., 2017), pose estimation for joint extraction [HRNet, Wang et al. (2019) backbone trained using the MMPose toolkit^1^] and finally 3D shape reconstruction [SMALR, Zuffi et al. (2018)]. The developed method is generalizable across 40 animal species spanning the *felidae, canidae, equidae, bovidae, and hippopotamidae* families, thereby encompassing many of the most commonly studied savanna species such as zebras, lions, hippos, and rhinos. Moreover, the derived shapes preserve surface texture information, enabling research on open-population individual animal identification using fully 3D-aware observations. Second is herd tracking and animals re-identification, which is the starting point for smart drone navigation: Figure 12 shows preliminary results of reliable (potentially *in-situ*) multi-animal animal tracking in close to real-time in 4k video streams, paving the way for all extensive in-field testing, integration with navigational components, and linking to animal-biometric measurement. The last will link to aspects such as side-based animal identification based on coat pattern, and behavioural fingerprinting or social recording of individuals within groups, extending the results in Figure 9b. GPU hardware such as the NVIDIA Jetson have been integrated into drones opening up real-world telemetry gathering and testing of the tracking platforms.

## 7 Conclusion and future work

The WildDrone contributions to computer vision and autonomous drones will be combined with new methods for conservation ecology to enable a range of cross-disciplinary scientific advances in technology and ecology. Drones are poised to revolutionize animal ecology and conservation by enabling practitioners to collect new types of data at unprecedented spatial and temporal resolution (Christie et al., 2016). Novel data streams generated by drones complement data from existing data collection methodologies, such as animal-borne sensors, human observation, and satellite imagery, to open doors to new scientific questions and allow researchers to answer these questions more precisely. For example, drone-based filming in combination with image-based tracking has enabled some of the first studies of the collective behaviour of wild animals in their natural environments (Hughey et al., 2018).

Communicating the developed technologies and conservational insights as well as sharing acquired (video) data will contribute to improved awareness of wildlife conservation. The measures acquired using the developed technology will provide new quantitative data and objective evidence to enable informed decision making by conservation biologists and policymakers. Moreover, the developed drone-based solutions will reduce the risk to humans engaged in wildlife conservation and monitoring activities. Drones will automate monotonous tasks such as detection, counting, and tracking wildlife, thus allowing human workers to focus on activities requiring more expertise and interpretation. The solutions will thus engage and empower end-users in the field of conservation with more reliable, efficient, and robust solutions.

In terms of future work, we observe that while a key goal of WildDrone is to demonstrate that drones are effective for observation tasks, other critical data modalities, like soundscapes and physical eDNA samples, remain difficult to gather at the necessary scale. For this reason, we are interested in the development of aerial, aquatic, and terrestrial robots for performing such sampling tasks. Such robot systems hold great potential for automating sampling tasks, but currently lack the autonomous capabilities to perform precise sampling across large areas.

## Data Availability

The raw data supporting the conclusions of this article will be made available by the authors, without undue reservation.

## Footnotes

1
https://github.com/open-mmlab/mmpose
